# Multi-Modality Imaging of Atheromatous Plaques in Peripheral Arterial Disease: Integrating Molecular and Imaging Markers

**DOI:** 10.3390/ijms241311123

**Published:** 2023-07-05

**Authors:** Xiaomeng Wang, Ying-Hwey Nai, Julian Gan, Cheryl Pei Ling Lian, Fraser Kirwan Ryan, Forest Su Lim Tan, Dexter Yak Seng Chan, Jun Jie Ng, Zhiwen Joseph Lo, Tze Tec Chong, Derek John Hausenloy

**Affiliations:** 1 Cardiovascular & Metabolic Disorders Program, Duke-National University of Singapore Medical School, Singapore 169857, Singapore; 2 Clinical Imaging Research Centre, Yong Loo Lin School of Medicine, National University of Singapore, Singapore 117599, Singapore; 3 Siemens Healthineers, Singapore 348615, Singapore; 4 Health and Social Sciences Cluster, Singapore Institute of Technology, Singapore 138683, Singapore; 5 Infocomm Technology Cluster, Singapore Institute of Technology, Singapore 138683, Singapore; ryan.kirwan@singaporetech.edu.sg (F.K.R.);; 6 Department of General Surgery, Khoo Teck Puat Hospital, Singapore 768828, Singapore; 7 Division of Vascular and Endovascular Surgery, Department of Cardiac, Thoracic and Vascular Surgery, National University Heart Centre, Singapore 119074, Singapore; 8 Department of Surgery, Yong Loo Lin School of Medicine, National University of Singapore, Singapore 119228, Singapore; 9 Vascular Surgery Service, Department of Surgery, Woodlands Health, Singapore 258499, Singapore; 10 Centre for Population Health Sciences, Lee Kong Chian School of Medicine, Nanyang Technological University, Singapore 308232, Singapore; 11 Department of Vascular Surgery, Singapore General Hospital, Singapore 168752, Singapore; 12 Surgical Academic Clinical Programme, Singapore General Hospital, Singapore 169608, Singapore; 13 Vascular SingHealth Duke-NUS Disease Centre, Singapore 168752, Singapore; 14 National Heart Research Institute Singapore, National Heart Centre, Singapore 169609, Singapore; 15 Yong Loo Lin School of Medicine, National University Singapore, Singapore 117597, Singapore; 16 The Hatter Cardiovascular Institute, University College London, London WC1E 6HX, UK

**Keywords:** peripheral artery disease (PAD), computed tomography (CT), magnetic resonance imaging (MRI), positron emission tomography (PET), single-photon emission computerized tomography (SPECT), molecular markers, vascular inflammation, plaque imaging

## Abstract

Peripheral artery disease (PAD) is a common and debilitating condition characterized by the narrowing of the limb arteries, primarily due to atherosclerosis. Non-invasive multi-modality imaging approaches using computed tomography (CT), magnetic resonance imaging (MRI), and nuclear imaging have emerged as valuable tools for assessing PAD atheromatous plaques and vessel walls. This review provides an overview of these different imaging techniques, their advantages, limitations, and recent advancements. In addition, this review highlights the importance of molecular markers, including those related to inflammation, endothelial dysfunction, and oxidative stress, in PAD pathophysiology. The potential of integrating molecular and imaging markers for an improved understanding of PAD is also discussed. Despite the promise of this integrative approach, there remain several challenges, including technical limitations in imaging modalities and the need for novel molecular marker discovery and validation. Addressing these challenges and embracing future directions in the field will be essential for maximizing the potential of molecular and imaging markers for improving PAD patient outcomes.

## 1. Introduction

Peripheral artery disease (PAD) is a common and debilitating condition characterized by the narrowing and obstruction of the antegrade flow of major systemic arteries other than those of the cerebral and coronary circulations, primarily due to the buildup of atherosclerotic plaques. PAD affects over 230 million people worldwide and is associated with significant morbidity, mortality, and reduced quality of life [1].

Several factors have been identified as major contributors to the development and progression of PAD. These include non-modifiable risk factors such as advanced age and genetic predisposition and modifiable ones such as smoking, diabetes, hypercholesterolemia, and hypertension. Lifestyle elements such as poor diet, physical inactivity, and obesity also play considerable roles [2,3].

Central to PAD’s pathogenesis is atherosclerosis, which is initiated when low-density lipoprotein (LDL) cholesterol accumulates within the artery walls, triggering an inflammatory response. This response leads to the recruitment of immune cells, primarily monocytes, that transform into macrophages, ingest the accumulated lipids, and become foam cells. Over time, the accumulation of these foam cells forms a lipid core within the arterial wall, which constitutes the atherosclerotic plaque [4].

Endothelial dysfunction is another key factor that promotes atherosclerosis in PAD. A healthy endothelium maintains vascular homeostasis by regulating vascular tone, cellular adhesion, thromboresistance, smooth muscle cell proliferation, and inflammation. However, in conditions such as hypertension, hypercholesterolemia, and diabetes, the endothelium becomes dysfunctional, promoting the adhesion and infiltration of inflammatory cells and the formation of atherosclerotic plaques [4].

As plaques grow and arterial stenosis worsens, blood flow to the affected limb is significantly reduced, leading to the clinical manifestation of PAD. Symptoms can range from asymptomatic disease to intermittent claudication—characterized by muscle pain or cramping during physical activity—to critical limb ischemia, where the blood flow is so poor that it leads to gangrene, necessitating limb amputation. Moreover, these plaques can become unstable and rupture, leading to acute limb ischemia, a severe and painful condition that requires immediate attention [3,5].

Early detection and accurate characterization of PAD are crucial for effective risk stratification, appropriate therapeutic intervention, and monitoring treatment response. In the landscape of diagnostic tools for peripheral arterial disease (PAD), traditional methods such as the ankle–brachial index (ABI) and pulse wave velocity (PWV) measurements have long served as reliable indicators. ABI, a straightforward, non-invasive tool, offers a quantifiable measure of the presence and severity of PAD. It accomplishes this by comparing the blood pressure in the ankle to the blood pressure in the arm, thus reflecting the adequacy of the blood flow [5,6]. However, while ABI can indicate the presence of PAD, it provides limited information about the precise location or extent of the disease.

On the other hand, PWV is a measurement of arterial stiffness, which has been recognized as a potential predictor of PAD. PWV measurements are obtained by capturing the velocity of the pressure waveform between two points along the arterial tree [7,8]. While effective in measuring arterial stiffness, this method does not offer direct insights into the anatomical characteristics or biological activities of atherosclerotic plaques. Although these traditional techniques are valuable for initial screening and diagnosis, they mainly provide functional information and lack the ability to visualize in-depth anatomical details, plaque composition, or molecular activities in PAD.

With the advent of advanced imaging technologies, the ability to non-invasively visualize and quantify PAD has significantly improved. Non-invasive medical imaging modalities, such as computed tomography (CT), magnetic resonance imaging (MRI), and nuclear imaging, such as single-photon emission computerized tomography (SPECT) and positron emission tomography (PET), have emerged as valuable tools for imaging PAD atheromatous plaques and vessel walls. These modalities provide detailed information on plaque burden, morphology, and composition and insights into vascular function and blood flow. Despite their higher cost and less widespread availability, these newer modalities hold the potential to revolutionize PAD diagnostics and prognostics. Furthermore, integrating molecular markers with imaging markers from these modalities can enhance our understanding of PAD pathophysiology and improve patient outcomes.

In this review, we will examine the applications of CT, MRI, and nuclear imaging in assessing PAD, focusing on the imaging markers derived from each modality and their potential correlations with molecular markers. We will also discuss the advantages and limitations of each imaging modality, the role of molecular markers in PAD pathophysiology, and the potential benefits of integrating molecular and imaging markers in the comprehensive assessment of PAD.

## 2. Method

An English-language literature search was conducted on PubMed, limited to articles published since 2000. The search was carried out in two stages: initially on 13 January 2023 and subsequently updated on 1 May 2023. A combination of title, abstract keywords (such as “peripheral artery disease” [Title/Abstract]), and Medical Subject Headings (MeSH, for instance, “Peripheral Arterial Disease” [Mesh]) were used to identify relevant studies that either evaluated or aimed to evaluate PAD using non-invasive medical imaging modalities or correlated imaging biomarkers with molecular markers of PAD.

In addition, reference lists from selected articles were examined to identify further applicable studies, and current practice guidelines were reviewed. Articles were selected for inclusion based on the quality of the research and its relevance to the review’s theme. For instance, studies that did not utilize non-invasive medical imaging modalities were excluded.

The search strategy yielded a total of 294 articles. From these, 14 pertaining to CT, 29 to MRI, 1 to SPECT, 7 to PET, and 6 studies discussing the correlation between imaging and molecular markers were selected for inclusion in this review.

## 3. Overview of Non-Invasive Medical Imaging Modalities

This section briefly overviews the primary non-invasive medical imaging modalities used for imaging PAD plaques and vessel walls: CT, MRI, and nuclear imaging. For each imaging modality, we discuss the basic principles, advantages, and limitations (Table 1).

### 3.1. Computed Tomography (CT)

CT imaging uses fan-beam X-rays and computer processing to generate cross-sectional images of the body’s internal structures [9]. It offers high spatial resolution, relatively fast acquisition time, and widespread availability, making it a valuable tool for PAD imaging. CT angiography (CTA) is commonly used to visualize arterial stenosis, calcification, and plaque morphology [10]. However, CT has some limitations, including exposure to ionizing radiation, the potential need for iodinated contrast agents (which may pose risks for patients with renal insufficiency), and limited soft-tissue contrast [11,12,13].

### 3.2. Magnetic Resonance Imaging (MRI)

MRI relies on nuclear proton magnetic resonance and the interaction of magnetic fields, radiofrequency pulses, and gradients to create detailed images of the body’s internal structures [14]. MRI offers excellent soft-tissue contrast, which allows for good visualization of vessel walls and plaque components [12]. Additionally, MR angiography (MRA) does not involve ionizing radiation and can be performed without imaging contrast, making it safe for repeated imaging. However, despite its superior tissue contrast and good resolution, MRI cannot identify calcified components in the vessel wall and plaque [15,16,17]. MRA also has limitations, such as longer scanning times, potential susceptibility to artifacts, and contraindications for certain patients (e.g., those with MR incompatible implants) [12].

### 3.3. Nuclear Imaging

Nuclear imaging involves injecting radiotracers to visualize the distribution and biochemical function of specific molecules or cells within the body. The two main types of nuclear imaging used in PAD assessment are positron emission tomography (PET) and single-photon emission computed tomography (SPECT). Nuclear imaging can visualize molecular and cellular processes in vivo by providing insights into plaque metabolism, inflammation, and neovascularization depending on the injected radiotracer [18,19]. However, PET and SPECT have lower spatial resolution than CT and MRI. Hybrid scanning modalities, such as PET/CT and PET/MRI, allow us to overcome this issue and combine the anatomical information provided by CT/MRI and functional information from nuclear imaging modalities. However, the limited availability of such hybrid scanning systems is a bottleneck for their wide applications. The current data suggest that there were 160 PET/MRI scanners worldwide in 2020 [20] and 1600 PET/CT scanners in the USA [21]. Nuclear imaging modalities also have limitations, including ionizing radiation, the risk of handling radioisotopes, and the need for specialized equipment and radiotracers [18].

In summary, CT, MRI, and nuclear imaging each offer unique advantages and limitations for PAD imaging. The choice of the most appropriate modality depends on the clinical context and the specific objectives of the assessment, such as plaque detection, characterization, or evaluation of treatment response. In the following sections, we will delve deeper into the applications and imaging markers associated with each modality and their potential integration with molecular markers for a comprehensive assessment of PAD.

## 4. Computed Tomography (CT)

In this section, we discuss the applications of CT in assessing PAD. With technological advances and accessibility, CTA has become a popular choice for PAD evaluation. CTA utilizes the emission and detection of X-rays as the patient passes through a gantry that rotates the X-rays in a 360° arc to generate three-dimensional data [22]. An intravenous bolus injection of iodinated contrast material enhances the visualization of blood vessels and distinguishes them from surrounding tissues. The scanning is timed to coincide with the arrival of the bolus of contrast material in the desired artery or vein [22].

### 4.1. CT for PAD Assessment

CTA is extensively employed to evaluate PAD, as it facilitates detailed visualization of the arterial lumen [23], detection of stenosis [24], and assessment of the extent and severity of the disease [25]. Early-generation helical CT scanners had a single detector that rotated around the gantry, resulting in long scan times and low-quality images, which were inadequate for accurately locating and assessing the severity of stenoses in the lower extremity vasculature. Subsequent generations of multidetector CT (MDCT) utilize multiple rows of smaller detectors to detect a wider coverage of X-rays as the patient moves through the gantry simultaneously, thus allowing for the rapid acquisition of images with higher spatial resolution than those obtained using earlier-generation scanners [26]. Currently, MDCTA is highly accurate for detecting hemodynamically significant lower limb stenoses and lesions [6,27]. For instance, modern 256-slice scanners can assess the degree of stenosis in the aortoiliac and lower limb occlusive disease with high sensitivity (93%) and specificity (92.7%) [25]. CTA could also detect peripheral arterial in-stent restenosis in excellent agreement (κ > 0.80) with color Doppler ultrasound, the current standard for detecting in-stent restenosis [28]. However, compared to digital subtraction angiography (DSA), the current gold standard for PAD diagnosis and evaluation, CTA has lower accuracy for assessing below-knee stenoses and lesions in the infrapopliteal segment [24,25].

Despite its limited diagnostic ability in the below-knee region, CTA has several advantages over DSA. Firstly, unlike catheter-based DSA, CTA is non-invasive and reduces catheter-associated patient risks and operator dependency. Additionally, although both DSA and CTA involve ionizing radiation, the radiation dose exposure of CTA is significantly lower than that of DSA [10]. Furthermore, CTA allows for improved evaluation of vessel walls and extravascular pathologies [25].

### 4.2. CT for Plaque Calcification Assessment

**Figure 1:**
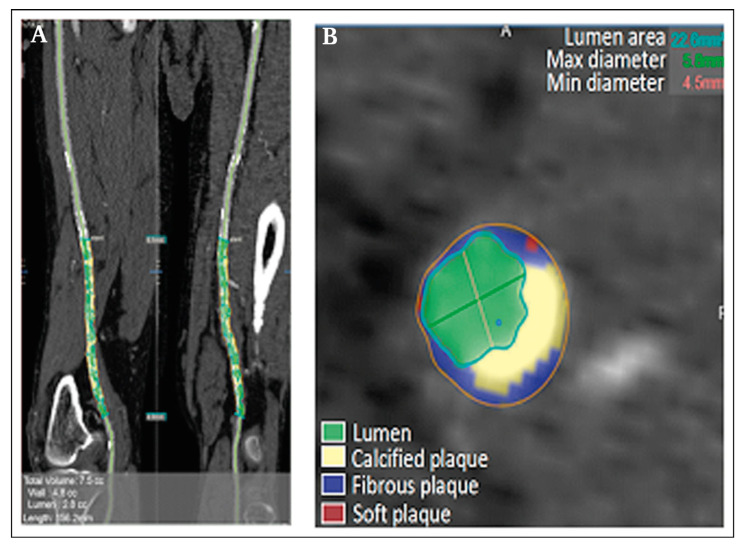
CT plaque tissue composition analysis of femoral artery plaque. (**A**) CTA images reconstructed in the volume-rendering mode; (**B**) automatic calculation of the calcified plaque volume within the cross-sectional image region of interest. The blue region indicates fibrous plaque; the green region indicates lumen; the red region indicates soft plaque; the yellow region indicates calcified plaque. Image reprinted from He et al. [31].

In addition to evaluating luminal narrowing, stenosis, and the extent of lesions, CT also provides information about vascular and plaque calcification, which is crucial for risk stratification in PAD (Figure 1). Patel et al., 2015, found that the burden of calcified plaque, but not soft or fibrocalcific plaque, was related to restenosis, reintervention, and amputation-free survival of PAD patients, highlighting the importance of CT plaque analysis in risk stratification for patients undergoing femoropopliteal endovascular procedures [29]. Kaladji et al., 2018, using patients from the STELLA and STELLA PTX registries, discovered that patients with severe vascular calcification (vascular calcification rate > 20%) were associated with early in-stent thrombosis (<1 month), while patients with no vascular calcification (vascular calcification rate < 1%) were associated with late stent thrombosis (6–24 months) [30]. He et al., 2019, found that among patients who received pre-operative CTA, those with a high calcified plaque burden had a higher risk for unfavorable outcomes, including in-stent restenosis, amputation, and mortality [31]. In the same year, Chang et al. determined that the lower limb calcification score was positively associated with acute thrombosis events in symptomatic PAD patients [32]. In 2021, Megale et al. found that in critical limb ischemia patients undergoing lower limb revascularization, pre-operative calcium scores of the aorta and operated limb arterial calcium scores were higher in patients who died within one and six months [33].

CT studies have also revealed that diabetes, a common underlying condition of PAD, is associated with increased lower limb vascular calcification [34,35]. In 2014, He et al. found that diabetes was associated with increased plaque incidence, particularly mixed plaque (plaques containing a calcified component, defined by an average attenuation of 60–100 HU). They also discovered that, in diabetic patients, lesions were more localized to the distal lower leg segments than in non-diabetic patients [35]. However, Mary et al. observed that, in type 2 diabetes mellitus patients, the use of metformin, but not other antidiabetic medications, was associated with a lower below-the-knee arterial calcification score, indicating a vascular protective effect of metformin [36].

Although CTA-derived vascular and plaque calcification aids in PAD risk stratification, the presence of vessel wall calcification often hinders accurate interpretation of peripheral artery CTA examinations. Streak and blooming artifacts caused by vessel wall calcification can lead to overestimating the vessel stenosis [37]. It has been found that the presence of arterial calcification decreases the clinical utility of CTA and compromises the accuracy of assessing hemodynamically significant stenosis [10,24]. One potential solution to this issue is dual-energy CT (DECT). By utilizing two different tube voltages, DECT can generate two datasets and, in theory, enable the extraction of iodine-contrast-only images without artifacts from bone, stent, and vascular calcification [38]. However, a DECT plaque subtraction simulation study using vessel phantoms still demonstrated an underestimation of lumen area in regions with calcified plaques, and this underestimation was more profound in smaller vessels [23].

### 4.3. Recent Advances and Future Perspectives

Recent advances in CT imaging, such as microCT, photon counting CT (PCCT), and artificial-intelligence-based image analysis, show promise in improving plaque characterization and assisting PAD diagnosis. An ex vivo study demonstrated that microCT might have better diagnostic performance among three lower limb plaque types (lipid-rich, fibrous, and calcified plaque) than conventional CTA [39]. Due to the micron-level high spatial resolution of microCT, more detailed information regarding vessel wall calcification can be obtained. Using microCT, Cahalane et al. observed a high prevalence of microcalcification (defined as calcium loci ≤ 65.4 × 10^3^ µm^3^) in both carotid and lower limb arteries. However, they found that weight-based extra-coronary calcium scores (ECCS) of both carotid and lower limb arteries had only weak positive correlations with the distribution of calcified particles (CPF, r_s_ = 0.422, *p* = 0.007) and microcalcifications (r_s_ = 0.361, *p* = 0.022) [40]. Although no studies associated the presence of microcalcification and calcified particles with adverse outcomes in PAD, these calcification morphologies were considered high-risk in the carotid and coronary artery disease [41,42,43]. Thus, to guide tailored treatment of high-risk plaques, it might be necessary to perform calcium scoring that distinguishes between critical calcification morphologies instead of simply providing a density-weighted score [40].

PCCT is a recently introduced technique in the field of cardiovascular CT. Traditional CT scanners employ energy-integrating detectors (EIDs), which measure the total energy deposited by X-ray in a detector pixel during a specific time frame. These EIDs do not differentiate between the number of photons and their individual energies. In contrast, PCCT utilizes a distinct type of detector called a photon-counting detector. These detectors can count individual X-ray photons and measure each photon’s energy, enabling enhanced image quality, superior contrast resolution, and the potential for reduced radiation dose to the patient [44,45]. In 2022, Si-Mohamed et al. published the first-in-human results comparing PCCT coronary CTA with conventional CTA. They discovered that PCCT coronary CTA exhibited improved image quality and diagnostic confidence compared to the energy-integrating dual-layer CT [46].

In addition to advances in CT technologies, developments in deep learning models can help with PAD diagnosis. In 2021, Dai et al. used a supervised convolutional neural network–parallel efficient network (p-EffNet) to classify CTA-derived lower limb artery segments according to the degree of stenosis, with DSA results used as a reference standard. The p-EffNet performed well in classifying both above-knee (91.5% accuracy, 90.2% sensitivity, and 97.7% specificity) and below-knee (90.9% accuracy, 91.3% sensitivity, and 95.2% specificity) arteries. Compared to radiologist readers, the p-EffNet had comparable accuracy and specificity but a lower sensitivity [47].

In summary, CT, specifically CTA, is a valuable tool for PAD imaging, providing crucial information on the extent and severity of arterial stenosis, plaque calcification, and morphology. Despite its limitations, ongoing technological advancements in CT imaging promise to improve its capabilities in characterizing plaques and assessing PAD.

## 5. Magnetic Resonance Imaging (MRI)

In this section, we will discuss the applications of MRI in assessing PAD. Unlike CT, which provides differential attenuation via the difference in atomic number between iodine contrast and soft tissue, magnetic resonance angiography (MRA) uses various tissue properties, including liquid flow, saturation, and relaxation times, to produce clear images with excellent soft-tissue contrast without ionizing radiation. However, despite its advantages, the application of MRA in routine PAD examinations remains limited, primarily due to the long exam duration and sensitivity to motion and other artifacts [10].

### 5.1. Contrast-Enhanced MRA

Among all the MRI-based methods, contrast-enhanced (CE)-MRA is the most used modality in PAD assessment because of its high accuracy and relatively short scan time compared to non-contrast MRA techniques [48,49]. Previous systematic reviews have shown that CE-MRA has similar or superior sensitivity and specificity for detecting lower limb artery stenosis compared to CTA or duplex sonography [48,50]. A gadolinium (Gd)-based contrast agent is the most common option for CE-MRI. Although a Gd-based contrast agent is safe for patients with normal renal function, it may cause a rare but incurable complication called nephrogenic systemic fibrosis (NSF) in patients with severely compromised renal function [49]. However, large studies have shown that the adverse reaction rate of a Gd contrast medium is lower than that of an iodinated contrast medium used in CT [51].

Iron-based nanoparticles, such as ultrasmall super-paramagnetic iron oxide (USPIO), are alternatives to Gd-based contrast agents for CE-MRI [52]. Ferumoxytol, a USPIO initially developed to treat iron deficiency anemia, has been employed as an MRI blood pool contrast agent due to its high T1 relaxivity and prolonged circulation time (mean intravascular half time of 15 h) [52]. Owing to its unique property, USPIO can function beyond anatomical delineation. Zheng et al., 2019, found that USPIO deposition in lower limb plaques was associated with plaque permeability, holding the potential to identify plaques susceptible to nanoparticle-delivered medication [53]. Additionally, since USPIO is eliminated by macrophages in the human body, it can be used to identify plaque and vessel wall inflammation. A previous study confirmed USPIO’s ability to capture carotid plaque inflammation by correlating plaque uptake of USPIO and ^18^F-fluorodeoxyglucose (FDG)-PET [54].

Besides contrast agents, advances in imaging sequences have also improved CE-MRA application. Time-resolved (TR) MRA is a dynamic technique that captures images at multiple time points during the passage of the contrast agent, providing anatomical and functional information [55]. The hemodynamic information from TR MRA has made it a valuable tool for evaluating flow-related pathologies, such as subclavian steal syndrome and arteriovenous malformation [56]. Previous studies have shown that TR MRA improves the diagnostic accuracy of distal vessel stenosis and enhances the diagnostic confidence of examiners [57,58].

### 5.2. Non-Contrast MRA

Time of flight (TOF) MRA is one of the earliest MRA techniques for peripheral artery assessment. The TOF technique generates high-resolution images of blood vessels by suppressing the signal from stationary tissues, allowing the inflow of hyperintense, unsuppressed blood without requiring intravenous contrast. However, TOF has only modest sensitivity and specificity and a prolonged scanning duration [59]. It may also be limited by slow flow, turbulence, and in-plane saturation effects [49]. Due to these limitations, TOF is not frequently used today.

Phase contrast (PC) MRA is another early MRA technique for peripheral artery assessment. PC MRA distinguishes blood flow from stationary tissue by measuring the phase difference between moving spins from blood and stationary spins from the background tissue [60]. Nowadays, the PC technique is primarily used to measure flow-related data. The so-called “4D flow” phase contrast techniques utilize a 3D sequence sensitized to flow in three directions, from which various parameters, such as wall shear stress, may be derived [61]. Combining morphological imaging from CE MRI with PC MRI with 3D velocity encoding, Galizia et al. found that patients with lower limb plaques demonstrated regionally increased peak systolic wall shear stress and enhanced wall shear stress eccentricity [62].

Electrocardiographic (ECG)-triggered fast spin echo (FSE) imaging utilizes ECG gating in combination with FSE pulse sequences to generate images of the blood vessels [63,64]. The ECG-FSE MRA technique synchronizes the FSE pulse sequence with the patient’s cardiac cycle using ECG gating. This method highlights the disparity between rapid blood flow in the arteries and sluggish blood flow in the veins during systole by utilizing the natural flow dephasing characteristics of spin echo sequences. The arteries are differentiated from the veins by subtracting the systolic images from the diastolic images. The ECG-FSE synchronization also helps to minimize motion artifacts caused by pulsatile blood flow and provides improved visualization of blood vessels, particularly in the lower extremities [65]. Due to the requirement of ECG gating and long acquisition time, arrhythmias and patient motion during the scan may cause artifacts and decrease image quality [49].

Balanced steady-state free precession (b-SSFP) MRA is another common non-contrast MRA modality in peripheral artery assessment. The b-SSFP MRA utilizes a fast gradient echo pulse sequence that achieves a steady-state magnetization with balanced gradients in all three spatial dimensions. This technique generates images with a high signal-to-noise ratio (SNR) and contrasts between blood vessels and surrounding tissues via the T2/T1 signal ratio, making it suitable for the PAD assessment [66].

A variant of b-SSFP, quiescent interval slice selective (QISS), utilizes a fast saturation recovery background suppression, timed together with inflow into the slice during the post-systolic quiet period [59,65]. Due to the rapid single-shot image acquisition and thin slices, QISS is less affected by patient motion and less sensitive to an in-plane saturation [67,68,69]. Comparison of QISS with CE-MRA [67] and CTA [70] confirmed its sensitivity and specificity for assessing peripheral artery stenosis.

### 5.3. MRI Techniques for Plaque Analysis

In recent decades, MRI has emerged as a valuable tool for the non-invasive characterization of peripheral artery plaques. It allows for the visualization and assessment of plaque composition, size, and morphology, providing reliable data for PAD evaluation and research applications. Imaging the vessel wall and plaque requires high spatial resolution, multiple contrasts, and good blood suppression techniques. An early study using a multi-slice FSE pulse sequence with fat presaturation showed good reliability for assessing plaque volume [71]. The limitations of 2D multi-slicing, in terms of coverage and resolution, were solved with the advent of high-resolution 3D FSE sequences, using parallel imaging acceleration techniques to achieve a reasonable scan acquisition time. The 3D Sampling Perfection with Application optimized Contrasts using different flip angle Evolutions (SPACE) sequence is a common alternative for assessing vessel walls and plaque. Due to high-resolution isotropic 3D imaging, SPACE enables superior vessel wall imaging with the aid of a multiplanar reconstruction [72]. In 2009, Mihai et al. compared T1-weighted SPACE (T1w-SPACE) with CE-MRA and confirmed the lumen area measurement results’ agreement between the two methods and the feasibility of measuring plaque [73]. Multiple contrasts can be achieved, for example, with T2 SPACE, which has a high time efficiency compared to 2D techniques [72]. This was further improved with the addition of “black-blood preparation” pulses to ensure more vascular dephasing for clearer vessel wall depiction. More complex pulses, such as DANTE (Delay Alternating with Nutation for Tailored Excitation), can be applied for this purpose. Combining DANTE black-blood preparation with 3D fast low-angle shot (FLASH), Xie et al. developed DANTE-FLASH, which allows for rapid isotropic-resolution imaging of the peripheral vessel wall at 3 Tesla (T) [74]. More MRI sequences have been discovered for their peripheral artery wall imaging capability in recent years, such as DESS (double echo steady state) [75,76]. Furthermore, using T2-weighted ultrashort echo time (UTE) sequences on a 7T MRI scanner, Roy et al. successfully differentiated ex vivo peripheral plaques with varying mechanical hardness [77].

**Figure 2:**
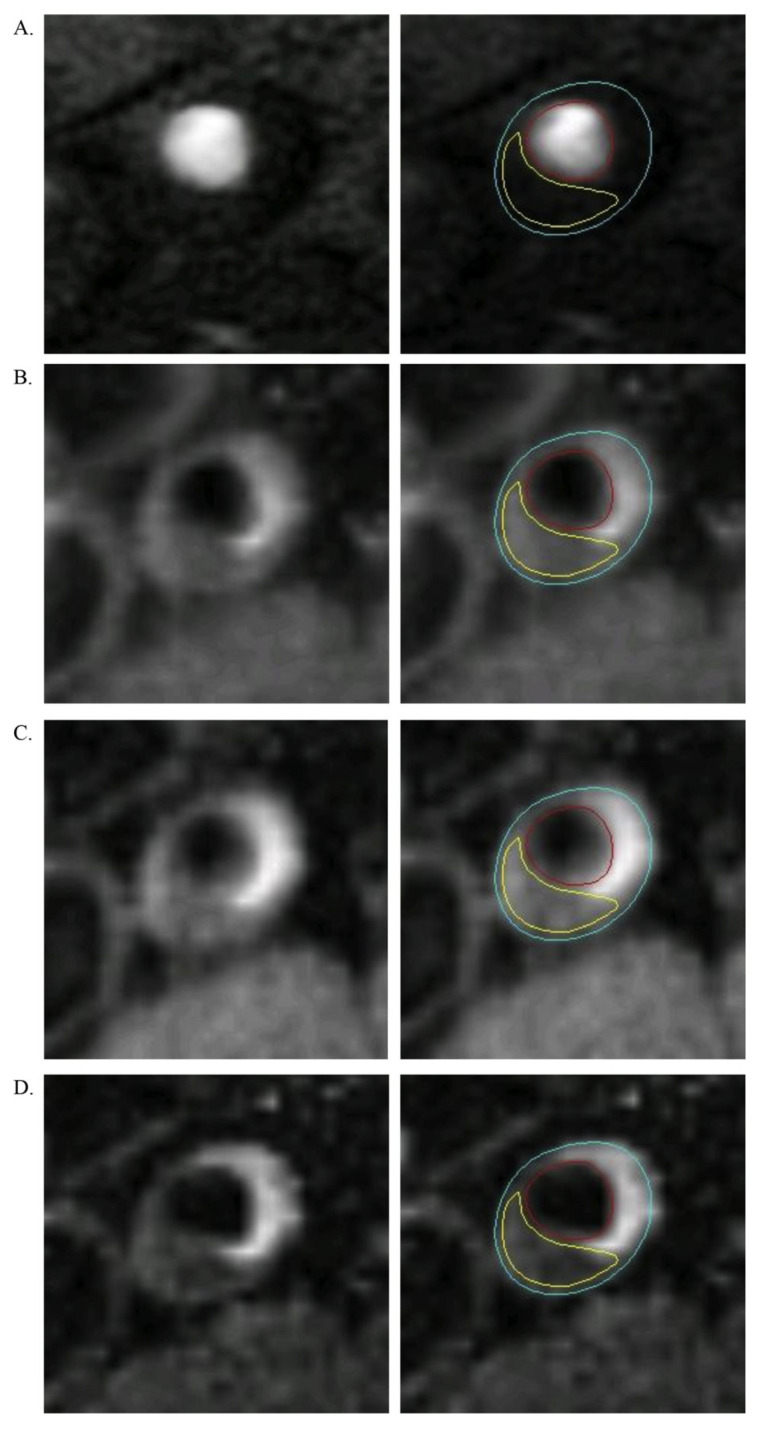
MRI presentation of superficial femoral artery plaque containing lipid-rich necrotic core. The imaging sequence is the time of flight (**A**); T1 (**B**); proton density (**C**); and T2 (**D**). The blue contour delineates the outer boundary of the SFA, the red contour delineates the lumen, and the yellow contour delineates the lipid-rich necrotic core. Image reprinted from Polonsky et al. [78].

In addition to structural measurements, the high soft-tissue contrast capability of MRI enables the extraction of plaque composition and high-risk features, such as lipid-rich necrotic core (LRNC) and intraplaque hemorrhage (IPH) (Figure 2). Unlike in CT, where tissue characterization is based on the absolute Hounsfield unit (HU) of voxels, tissue types in MRI are identified based on signal intensities relative to the surrounding muscles (e.g., sartorius muscle). According to Polonsky et al., LRNC without hemorrhage is hypointense on T2-weighted images, isointense or slightly hyperintense on T1-weighted images, and isointense on proton-density-weighted and TOF images. IPH is hyperintense on TOF and T1-weighted images and hyperintense/hypointense (depending on the stage of hemorrhage) on T2-weighted and proton-density-weighted images [78].

A correlation study between PET/CT and MRI demonstrated that peripheral artery plaques with MRI-derived LRNC exhibit greater inflammation than fibrous or calcified plaques [79]. However, researchers also discovered that compared to carotid plaques, femoral artery plaques tend to have smaller necrotic cores and less hemorrhage [80]. In 2014, Polonsky et al. found that among 302 adult PAD patients, 22.4% had proximal SFA plaques containing LRNC, while only one patient had plaques with IPH [78]. In 2017, McDermott et al. reported that among PAD patients, LRNC in the SFA plaques was associated with a higher risk of clinical PAD events (lower extremity amputation, critical limb ischemia, ABI decline > 0.15, and revascularization) at 47-months follow-up, and this association was independent of ABI [81].

### 5.4. MRI for PAD Clinical Studies

Due to the efficacy of objective plaque volume evaluation, MRI has been used in observational studies to evaluate peripheral plaque volume, morphology, and vessel stenosis. Previous studies have found that MRI-derived superficial femoral artery (SFA) stenosis, occlusion, and plaque burden were associated with impaired ABI and exercise tests, such as the 6 min walking test, and mobility loss [82,83,84,85,86]. In 2014, Weir-McCall et al. found that in symptomatic PAD patients, patients’ ABI had a significant negative correlation with MRA-derived whole-body standardized atheroma score, primarily due to the significant negative correlation between ABI and standardized atheroma score in the iliofemoral vessels [87]. In 2016, Bosch et al. discovered that the mean MRA stenosis class (i.e., average stenosis severity visually scored over 27 standardized segments across the body) was a significant independent predictor for all-cause mortality [88]. They also found that MRA-derived PAD stenosis correlated well with distal aortic stiffness but to a lesser extent with proximal aorta stiffness [89]. Besides symptomatic PAD patients, MRI has also been used to examine peripheral arterial plaques in non-symptomatic subjects. In 2018, Weir-McCall et al. found that compared to Western Europeans, South Asians have a lower iliofemoral plaque burden [90]. In the same year, Han et al. discovered that subclinical femoral artery atherosclerosis is prevalent in the elderly population [75], particularly in the left femoral artery and segments of the common femoral artery and popliteal artery. However, there was no significant difference in ABI between subjects with and without atherosclerotic plaques [91].

Moreover, MRI has also been employed in randomized clinical trials to evaluate the effect of medications on peripheral artery plaque progression. In 2011, using MRI-measured SFA wall volume change as the primary endpoint, West et al. found that statin initiation with or without ezetimibe in statin-naïve patients halted the progression of peripheral artery atherosclerosis. However, researchers found that in patients previously on statins, peripheral artery atherosclerosis progressed when ezetimibe was added to the treatment scheme [92]. In 2019, Russell et al. used 3T black-blood MRI to measure SFA plaque progression in symptomatic PAD patients taking canakinumab, an interleukin-1β (IL-1β) neutralizing antibody, and a placebo control. Although canakinumab may have improved maximum and pain-free walking distance in PAD patients, researchers found no evidence of plaque progression in the SFA in either placebo-treated or canakinumab-treated patients at 3 or 12 months [93].

### 5.5. AI Application in MRI PAD Assessment

Accurate peripheral artery segmentation is crucial for diagnosing and monitoring PAD, assessing plaque burden, and planning therapeutic interventions. However, manual segmentation is time-consuming, labor-intensive, and prone to inter- and intra-observer variabilities. In recent years, advances in computational capabilities and AI have shown great promise in MR image analysis, particularly for segmenting peripheral artery structures. In 2015, Chen et al. proposed a semi-automatic algorithm to generate accurate femoral artery lumen and outer wall boundaries from 3D black-blood MR images with minimal user interaction, using only 1% of the time required for manual segmentation [94]. In the same year, Ukwatta et al. proposed another semi-automatic algorithm-coupled continuous max-flow (CCMF) model, to jointly segment the femoral artery’s lumen and outer wall surface. Their model demonstrated both high accuracy (Dice similarity coefficients ≥ 87% for both the lumen and outer wall surfaces) and high reproducibility (intra-class correlation coefficient of 0.95 for generating vessel wall area) [95]. Mistelbauer et al. found that the application of semi-automatic lower limb vessel segmentation tools to clinical workflow enabled expert physicians to readily identify all clinically relevant lower extremity arteries with an average sensitivity of 92.9%, an average specificity, and an overall accuracy of 99.9% while saving 39% of the time [96]. In 2020, Hippe et al. developed a fully automated deep-learning-based algorithm called Fully Automated and Robust Analysis Technique for Popliteal Artery Evaluation (FRAPPE) to segment and quantify the popliteal artery wall for the Osteoarthritis Initiative (https://nda.nih.gov/oai/). After applying confidence weighting, Hippe et al. showed that FRAPPE could yield satisfactory scan–rescan coefficients of variation statistics for popliteal artery morphology measurements while significantly reducing the time needed for the segmentation [97].

In summary, MRI is a valuable tool for PAD imaging, providing detailed information on luminal stenosis, plaque composition, and vessel wall characteristics. Despite its limitations, ongoing technological advancements in MRI promise to improve its capabilities in characterizing plaques and assessing PAD. Furthermore, integrating AI and machine learning in MRI analysis will likely streamline the diagnostic process, enhance accuracy, and reduce the time and labor required for segmentation and assessment.

## 6. Nuclear Imaging

Nuclear imaging has been employed to evaluate tissue perfusion, viability, and functionality [98]. Although limited by spatial resolution and unable to provide as much anatomical information as CT and MRI, nuclear imaging modalities can depict molecular and cellular processes, yielding valuable pathophysiological information. In this section, we discuss the applications of nuclear imaging in assessing PAD, focusing on SPECT and PET, as well as their specific imaging markers and recent advances.

### 6.1. SPECT for PAD Assessment

SPECT has been used in clinical settings for decades for various cardiovascular applications, such as myocardial perfusion assessment, and has recently emerged as an approach for PAD pathophysiology [18]. SPECT enables 3D functional imaging of targeted radiolabeled probes through non-invasive detection of emitted gamma rays. Thallium-201 (^201^Tl) and technetium-99m (^99m^Tc)-based radiotracers are commonly employed for SPECT. The most prevalent application of SPECT in PAD assessment is skeletal muscle perfusion, using ^201^Tl and [^99m^Tc] tetrofosmin [18]. SPECT can also be used to detect inflammatory signals from PAD complications, such as lower limb infection and osteomyelitis, using [^99m^Tc] diphosphonate, [^99m^Tc] anti-granulocyte antibodies, and indium-111 (^111^In)-labeled leukocytes [18,99,100,101]. However, the application of SPECT in plaque imaging is very limited, potentially due to SPECT’s significantly lower spatial resolution compared to PET. In 2014, Johnson et al. used a ^99m^Tc-labeled antibody targeting the receptor for advanced glycated end-products (RAGE), a mediator for atherosclerosis plaque initiation and progression, to investigate the utility of RAGE-directed SPECT imaging in locating atheroma plaques in hyperlipidemic pig models. Johnson et al. discovered that focal vascular uptake of a radiotracer visualized on SPECT scans corresponded to AHA class III/IV lesions in the coronary and carotid vessels. Tracer uptake in hind limbs corresponded to RAGE staining of small arteries in the muscle sections. Their study supported the critical role of RAGE in PAD as a potential imaging target [102].

### 6.2. PET for PAD Assessment

Positron emission tomography (PET), another nuclear imaging technique, detects pairs of gamma rays emitted by positron-emitting radiotracers and offers visualization of molecular and cellular processes in PAD. Compared to SPECT, PET is limited by higher costs and availability. However, PET boasts higher spatial resolution and sensitivity and provides quantitative information on the radiotracer uptake [103]. Like SPECT, PET can also measure muscle perfusion and detect complications such as infection, osteomyelitis, Charcot foot, and peripheral neuropathy [18]. PET radiotracers, such as ^15^O-water, ^13^N-ammonia, and ^18^F-FDG, are commonly used in PAD-related applications of the PET [18,98].

**Figure 3:**
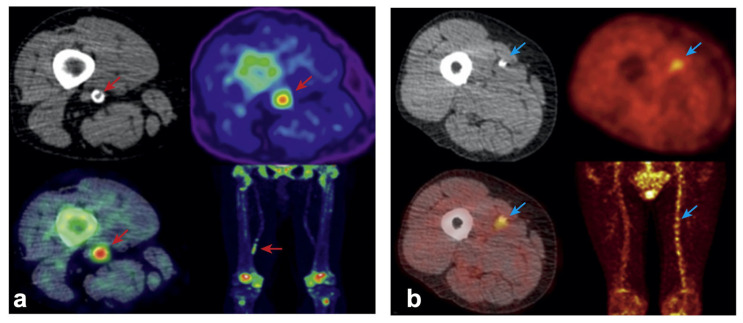
(**a**) Non-contrast CT (top left) with a rim of calcification of the vessel, ^18^F-NaF PET (top right), and fused ^18^F-NaF PET/CT (bottom left) of the superficial femoral artery (red arrow) at the level of the adductor canal, demonstrating significant vessel uptake in a patient with claudication at 300 yards. In addition, prominent uptake is seen in the vessel at the same level on the coronal image (bottom right); (**b**) non-contrast CT (top left) with calcification of the vessel, ^18^F-FDG PET (top right), and fused non-contrast CT (bottom left) of the superficial femoral artery (blue arrow) at the level mid-thigh, demonstrating significant vessel uptake in a patient with tissue loss in the left leg. In addition, prominent uptake is seen across the whole vessel coronal image (bottom right), compared with the contralateral leg. CT = computed tomography; ^18^F-FDG = ^18^F-fluorodeoxyglucose; ^18^F-NaF = ^18^F-sodium fluoride; PET = positron emission tomography. This image is reprinted from Chowdhury et al. with modification [106].

Among all the radiotracers, ^18^F-fluorodeoxyglucose (^18^F-FDG) is the most widely used for imaging atherosclerosis. Inflammatory cells, such as activated macrophages and lymphocytes, exhibit increased glucose metabolism and hence ^18^F-FDG uptake. This uptake, commonly measured as the target-to-background ratio (TBR), is employed to measure plaque inflammation [104]. In PAD patients, high-risk vulnerable and ruptured plaques exhibit increased ^18^F-FDG uptake (Figure 3b) [79,105]. Researchers also found that in PAD patients who underwent lower limb percutaneous transluminal angioplasty (PTA), those with greater femoral arterial ^18^F-FDG uptake had a higher risk of restenosis [106]. Beyond the context of PAD, ^18^F-FDG PET can also be used to evaluate arterial inflammation in subclinical or non-atherosclerotic patients. In patients with type 2 diabetes, researchers found that lower limb arterial uptake of ^18^F-FDG was positively associated with increased vessel stiffness [107]. Researchers also found that with increasing age, subjects, both healthy and with PAD, tend to have increased uptake of ^18^F-FDG in lower limb arteries, indicating a positive correlation between age and lower limb vascular inflammation [108,109,110,111]. In 2019, using participants from the Progression of Early Subclinical Atherosclerosis (PESA, NCT01410318) study, Fernández-Friera et al. found that peripheral arterial inflammation is highly prevalent in middle-aged individuals with subclinical atherosclerosis, and 61.5% of ^18^F-FDG uptake was detected in plaque-free arterial segments. They also found that among these subclinical subjects, those with arterial inflammation had a greater plaque burden [112].

Due to the high sensitivity of ^18^F-FDG PET in capturing arterial inflammation, it has also been used as an endpoint measurement for clinical interventions. In 2008, Lee et al. found that, in healthy subjects, vascular ^18^F-FDG uptake was reversed in response to atherogenic risk-reducing lifestyle interventions, and the magnitude of improvement correlated with increases in plasma high-density lipoprotein (HDL) levels [113]. In 2010, Ishii et al. found that six months of treatment with atorvastatin 20 mg, but not 5 mg, was associated with a significant reduction in ascending aorta and femoral artery ^18^F-FDG uptake in Japanese adults with dyslipidemia [114]. In 2021, Jiang et al. found that sonodynamic therapy (SDT), a novel non-invasive macrophage-targeted anti-inflammatory regimen for atherosclerosis, reduced ^18^F-FDG uptake in the femoropopliteal artery within 30 days. They also found that SDT increased patients’ ABI, while no correlation between the change in ABI and ^18^F-FDG uptake changes during the treatment course was made in the study [115].

However, despite the widespread application of ^18^F-FDG PET in detecting vascular inflammation in PAD, some issues still need to be addressed regarding the evaluation of PAD plaques using ^18^F-FDG PET. In 2017, Dregely et al. compared the SFA plaque histology with imaging results from ^18^F-FDG PET and MRI. These researchers found that the MRI images successfully identified the fibrous nature of scanned plaques, but they failed to correlate ^18^F-FDG PET-identified cellular inflammation with histological signs of macrophage activity. Dregely et al. suggested their results indicated a different pathophysiology between carotid/coronary plaques and SFA plaques, which have reduced macrophage activity [116]. Moreover, since FDG is a glucose analog, plaque uptake of ^18^F-FDG can be affected by baseline blood glucose levels and various conditions affecting tissue glucose uptake, such as diabetes and medications. Previous studies have found that ^18^F-FDG PET could underestimate vascular inflammation in diabetic patients with poorly controlled blood glucose [117]. Since diabetes is an important trigger for PAD, this drawback raises concerns about applying ^18^F-FDG PET in PAD assessment. To overcome this limitation, Gallium-68-labeled DOTA-(Tyr^3^)-octreotate (^68^Ga-DOTATATE), which binds to somatostatin receptor subtype-2 (SSTR2) on activated macrophages, was discovered as an alternative tracer for imaging atherosclerotic inflammation. A previous study demonstrated that ^68^Ga-DOTATATE had excellent macrophage specificity and greater power to discriminate high-risk versus low-risk coronary lesions than ^18^F-FDG [118]. The applicability of ^68^Ga-DOTATATE PET in measuring peripheral plaque inflammation is still pending further investigation.

In recent years, more radiotracers have been involved in PAD plaque imaging, broadening the application of PET. Due to its high affinity to calcium phosphate minerals, ^18^F sodium fluoride (^18^F-NaF) can be used to detect calcification, including microcalcification, in plaque development (Figure 3a) [119]. In 2010, Derlin et al. confirmed the feasibility of imaging femoral artery calcification [120]. Recent studies showed that peripheral plaque calcification is associated with cardiovascular risk factors and increased risk for post-PTA restenosis [106,121]. Acetate is a crucial substrate for fatty acid synthesis, and ^11^C-acetate has been used for monitoring metabolic activity in tumors and myocardium [122,123]. Derlin et al. demonstrated peripheral arterial uptake of ^11^C-acetate, indicating the feasibility of using ^11^C-acetate to monitor fatty acid synthesis in plaque and vessel walls [124].

In summary, nuclear imaging, specifically SPECT and PET technologies, offers valuable insights into PAD at the molecular and cellular levels. Despite their limitations, ongoing advancements in nuclear imaging promise to improve their capabilities in characterizing plaques and assessing PAD. By using novel radiotracers and refining imaging technologies, these methods will continue to enhance our ability to characterize atherosclerotic plaques and assess the pathophysiology of PAD.

## 7. Non-Invasive Perfusion Imaging in PAD

Microvasculature holds a critical role in the pathogenesis of PAD, particularly in relation to tissue ischemia, ulceration, and gangrene. Traditional vascular imaging methods such as CTA and MRA facilitate the assessment of patency and stenosis of major vessels. However, these techniques are incapable of measuring local perfusion in the lower extremities. Recently, perfusion imaging has surfaced as a potential remedy to this limitation. In this section, we will provide a concise overview of the application of CT, MRI, and nuclear imaging modalities in perfusion imaging. An in-depth exploration of perfusion imaging techniques and their clinical applications can be found in other comprehensive reviews [18,125,126,127].

CT perfusion, enabled by technological advancements and dedicated software, allows for the qualitative and quantitative evaluation of tissue perfusion in PAD patients. This technique measures changes in tissue density following the intravenous administration of an iodinated contrast medium, providing estimates of perfusion parameters such as blood flow, blood volume, mean transit time, and permeability surface [128]. Early studies have demonstrated the feasibility and reproducibility of CT perfusion for assessing tissue perfusion, with successful revascularization leading to significant increases in the blood flow [126]. Moreover, CT perfusion has been shown to have a prognostic role, with lower post-PTA perfusion parameters associated with poor outcomes [129]. The technique also correlates well with clinical and hemodynamic parameters [130]. Despite its advantages, including rapid examination time and the ability to provide information about lower limb hypoperfusion, CT perfusion has downsides, such as patient radiation exposure and the need for an iodinated contrast [126].

MRI techniques, including dynamic-contrast enhanced (DCE) MRI, blood-oxygenation-level-dependent (BOLD) MRI, arterial spin labeling (ASL), and intravoxel incoherent motion (IVIM) MRI, offer various methods for assessing microcirculation and perfusion in PAD patients. DCE-MRI uses gadolinium-based contrast to calculate perfusion parameters, and it has been used to measure skeletal muscle perfusion and assess the outcome of PTA. BOLD-MRI, a standard technique for functional MRI, has been used to compare calf muscle perfusion in PAD patients and healthy individuals, showing significant differences in perfusion parameters. ASL, a non-contrast technique, measures tissue blood flow using arterial water protons labeled by radiofrequency pulses, and it has been used to measure blood flow in PAD patients and healthy individuals, showing significant differences in peak exercise calf perfusion. IVIM-MRI assesses the translational motions inside a voxel, including molecular diffusion of water and microperfusion in the capillary network, and it has been used to assess quantitative changes in lower limb muscle perfusion and evaluate foot microperfusion in PAD patients. Despite their advantages, including the ability to perform examinations without radiation and, in some cases, without contrast agents, these MRI techniques are time-consuming, require patient cooperation, and require specific equipment and specialized personnel [125,126].

Nuclear imaging modalities are also adopted for the perfusion imaging of PAD. Planar scintigraphy and SPECT/CT have been used since the 1940s for evaluating lower extremity perfusion in PAD patients. ^201^Tl became widely used due to its redistribution characteristics, allowing for evaluating skeletal muscle perfusion under stress and rest conditions with a single injection [18]. Studies have shown that ^201^Tl scintigraphy can detect skeletal muscle perfusion defects in asymptomatic diabetic patients with normal ABIs [131]. More recent work has shown that hybrid ^201^Tl SPECT/CT imaging can detect changes in regional skeletal muscle perfusion associated with active angiogenesis and arteriogenesis [18].

[^99m^Tc]sestamibi and [^99m^Tc]tetrofosmin are the most commonly used radiotracers for evaluating lower extremity skeletal muscle perfusion [132]. These radiotracers have advantages over ^201^Tl due to a shorter half-life and higher energy emission, enhancing count detection, reducing scatter, and resulting in less attenuation on gamma cameras. Studies using these radiotracers have effectively detected manifestations of PAD, including abnormalities in skeletal muscle perfusion associated with upstream arterial occlusions [18].

PET imaging techniques have also been used for assessing lower extremity skeletal muscle perfusion in PAD. Early studies using [^15^C]CO_2_ and ^15^O_2_ inhalation revealed asymmetrical patterns of calf muscle blood flow in PAD patients during ankle flexion. Subsequent studies using [^15^O]H_2_O PET imaging successfully quantified absolute calf muscle perfusion at rest, during exercise, and after infusion of prostaglandin E_1_ in healthy subjects and PAD patients. PET-derived measures of muscle blood flow have been shown to correlate closely with traditional blood flow assessment techniques. Ongoing developments with modern PET imaging systems are expected to bring additional opportunities for non-invasive imaging of PAD and improve the quantitative assessment of lower extremity perfusion in the setting of limb ischemia [18].

In summary, while the role of microvasculature in PAD, especially in relation to tissue ischemia, ulceration, and gangrene, is increasingly recognized, the capacity of current imaging modalities to fully capture these intricacies remains limited. Perfusion imaging may complement traditional imaging techniques in certain scenarios, particularly for assessing the microvasculature. However, more research is needed to fully understand microvasculature’s role in PAD and optimize perfusion imaging in clinical practice.

## 8. Molecular Markers in PAD and Their Correlations with Imaging Markers

This section discusses various molecular markers involved in PAD, focusing on their correlation with imaging markers in non-coronary artery disease (CAD) imaging studies (Table 2). A more detailed and comprehensive review of molecular markers and their roles in PAD pathophysiology, diagnosis, and prognosis can be found elsewhere [133].

### 8.1. Inflammatory Markers

Inflammation plays a critical role in the initiation and progression of PAD. Several inflammatory markers have been identified as potential PAD severity and prognosis indicators. Some inflammation-indicating plasma molecular biomarkers, such as interleukin-6 (IL-6), matrix metalloproteinases (MMPs), and lipoprotein-associated phospholipase A_2_ (Lp-PLA_2_), and anti-inflammatory biomarker adiponectin are associated with various non-invasive imaging markers.

IL-6 is a common inflammatory marker for PAD. As a pro-inflammatory cytokine, IL-6 can be produced by various cells, such as vascular smooth muscle cells and endothelial cells, leading to various downstream effects, including CRP production. Some researchers believe IL-6 is more specific to vascular inflammation than CRP [134]. Previous studies have found higher IL-6 levels associated with the incidence of PAD and PAD severity [135,136,137,138,139]. Supporting the role of IL-6 in PAD diagnosis and risk stratification, Chen et al. found that patients with a higher grade of CT-derived peripheral artery stenosis had higher IL-6 levels than those with lower stenosis grades (Table 2) [140].

Matrix metalloproteinases (MMPs) are a group of enzymes that require zinc to break down extracellular matrix proteins. They are produced by various inflammatory cells that participate in both physiological and pathological processes. In atherosclerosis, MMP dysregulation is linked to leukocyte infiltration, vascular smooth muscle cell (VSMC) migration, and plaque formation [133]. Studies have shown that PAD patients have increased MMP-1, -2, -3, -7, -9, -10, -12, -13, suggesting a potential role in the PAD diagnosis [137,141,142,143]. In 2009, Rudd et al. found that in atherosclerosis patients and high-risk subjects, serum MMP-3 levels were associated with ^18^F-FDG uptake, measured by the TBR of ^18^F-FDG PET, in the descending aorta, while MMP-9 levels were associated with carotid TBR [144]. Wu et al. confirmed the association between MMP-9 levels and carotid TBR in hyperlipidemic atherosclerosis patients. They found that atorvastatin-treatment-induced carotid TBR change was correlated with changes in MMP-9 levels [145]. Besides circulating MMP-9 levels, a histology study found that carotid plaque MMP-9 content was also associated with ^18^F-FDG PET signal (Table 2) [146].

Lp-PLA_2_ is another inflammatory protein produced by inflammatory cells to hydrolyze oxidized phospholipids on the surface of LDL particles. Previous studies have found that increased Lp-PLA_2_ mass and activity predict an increased risk for PAD incidence and low ABI [147,148]. Using participants from the dal-PLAQUE trial, who were taking dalcetrapib (a cholesteryl ester transfer protein inhibitor), Duivenvoorden et al. found that Lp-PLA_2_ mass correlated with aorta TBR at most diseased segments (TBR_mds_), but this correlation disappeared at the 3-month follow-up (Table 2) [149].

In addition to IL-6, MMPs, and Lp-PLA_2_, other inflammatory markers are involved in PAD evaluation. C-reactive protein, a well-established marker of systemic inflammation, is the most studied molecular marker for PAD diagnosis and prognosis. Circulating CRP levels are increased in PAD patients compared to healthy subjects and are associated with PAD severity [135,150,151,152,153]. Elevated CRP levels in PAD patients are associated with an increased risk of major adverse cardiovascular events (MACE) and mortality [154,155]. However, although CRP levels are associated with greater CT-derived plaque burden and calcification in CAD, the relationship between CRP levels and imaging characteristics in other vascular beds remains understudied [156]. In 2009, Rudd et al. conducted a correlational study between CRP levels and ^18^F-FDG uptake in carotid arteries, aortas, and peripheral arteries but failed to find any significant correlation [144]. In 2020, Bueno et al. found that symptomatic carotid artery disease patients had higher ^18^F-FDG uptake and circulating CRP than asymptomatic patients, but a direct correlation between ^18^F-FDG uptake and CRP levels was not presented [157]. Other inflammation-indicating biomarkers, such as IL-8, pentraxin-3 (PTX-3), neutrophil gelatinase-associated lipocalin (NGAL), calprotectin, or tumor necrosis factor (TNF)-α, have also shown their potential in PAD diagnosis, among which PTX-3 and TNF-α were also associated with PAD severity [133,135,143,151]. Future studies are still needed to illustrate the relationship between these inflammatory markers and imaging markers of PAD.

In addition to pro-inflammatory factors, adiponectin, an anti-inflammatory factor, also plays a role in PAD evaluation. Urbonaviciene et al. found that higher adiponectin levels reduced the risk for future cardiovascular events in PAD patients, suggesting a potential vascular protective effect [158]. In agreement with this conclusion, Rudd et al. found that circulating adiponectin levels were negatively correlated with the degree of descending aorta inflammation measured by ^18^F-FDG PET (Table 2) [144].

### 8.2. Endothelial Dysfunction and Oxidative Stress Markers

Endothelial dysfunction is a key factor in the development and progression of PAD. Several biomarkers have been associated with endothelial dysfunction in PAD, including adhesion molecules (intercellular adhesion molecule 1 [ICAM-1] and vascular cell adhesion protein 1 [VCAM-1]) and selectins (E-selectins and P-selectins) [159]. PAD patients have been found to have elevated levels of endothelial dysfunction markers compared to healthy subjects [133].

However, the correlation between imaging markers and these endothelial dysfunction markers remains unclear. In 2012, Wu et al. found that in hyperlipidemic atherosclerosis patients undergoing statin therapy, both E-selectin levels and carotid ^18^F-FDG PET TBR decreased through the treatment, but no association was found between the two reductions [145]. Similarly, in the dal-PLAQUE trial, soluble P-selectin, soluble E-selectin, soluble ICAM-1, and soluble VCAM-1 levels were not associated with either aorta or carotid TBR_mds_ [149]. Future studies are still needed to illustrate the relationship between endothelial dysfunction markers and imaging markers of PAD.

Oxidative stress is a crucial contributor to endothelial dysfunction and atherosclerotic plaque formation in PAD. Myeloperoxidase (MPO), a key oxidative stress marker of PAD, is a peroxidase that catalyzes the formation of potent oxidants by neutrophils and macrophages, inducing low-density lipoprotein (LDL) oxidation in blood with high antioxidant status [160]. In patients with chronic limb-threatening ischemia, elevated MPO levels predict an increased risk for cardiovascular events [155]. In the dal-PLAQUE trial, Duivenvoorden et al. found that baseline MPO levels positively correlated with baseline carotid TBR_mds_, and this correlation remained significant at the 3-month follow-up (Table 2) [149]. Several other markers of oxidative stress, including oxidized LDL (oxLDL) and malondialdehyde (MDA), have also shown their potential in the PAD diagnosis [4]. Future studies are still needed to illustrate the relationship between these oxidative stress biomarkers and PAD imaging markers.

### 8.3. Other Biomarkers

Apart from the aforementioned categories, other biomarkers have been linked to PAD. Angiogenesis factors, such as vascular endothelial growth factor (VEGF) and hypoxia-inducible factor (HIF)-1α, promote capillary network growth in response to local hypoxia in the ischemic limb of the PAD [161]. In 2015, Chen et al. discovered that serum VEGF-C levels increased in patients with higher grades of CT-derived peripheral artery stenosis (Table 2) [140].

Considering the role of vascular calcification in PAD evaluation, vascular calcification markers have also garnered attention. Matrix Gla-Protein (MGP) is a vitamin-K-dependent vascular calcification inhibitor, and inactive MGP is associated with arterial stiffness and increased risk for PAD [162,163,164]. Osteonectin is a non-collagenous calcium-binding glycoprotein of bone matrix involved in developing and mineralizing bone tissue [165]. Recent in vivo and ex vivo studies discovered that oxLDL could induce osteonectin production in VSMCs and that osteonectin levels increased in atherosclerotic and calcified vessels, suggesting the potential role of osteonectin as a vascular calcification factor [166,167]. In 2020, Zwakenberg et al. assessed the vascular calcification status of participants from the Second Manifestations of ARTerial disease (SMART) study and the Hoorn Diabetes Care System (DCS) cohort using CT. Femoral calcification was found in 77% of the participants, of whom 38% had intimal arterial calcification (IAC) and 28% had medial arterial calcification (MAC). After correlating calcification phenotypes with plasma biomarkers, researchers found that inactive MGP levels were associated with an increased prevalence of MAC. In contrast, increased osteonectin levels were associated with decreased MAC prevalence compared to IAC, suggesting a different pathophysiology behind these two calcification phenotypes (Table 2) [168].

**Table 2:** Studies correlating molecular markers of PAD with the aorta, carotid, and lower limb peripheral artery imaging markers.

Imaging Modality	Study Population (n)	Molecular Biomarker	Location	Biomarker Source	Conclusion	Ref.
^18^F-FDG-PET	Patients with either vascular disease or at least 3 cardiovascular risk factors (41)	MMP-3, MMP-9, adiponectin	Aorta, Carotid	Serum	Subjects with the highest levels of FDG uptake also had the greatest concentrations of inflammatory biomarkers (descending aorta TBR vs. MMP-3; carotid TBR vs. MMP-9). Atheroprotective biomarker adiponectin was negatively correlated with the degree of descending aorta inflammation (TBR).	[144]
^18^F-FDG-PET	Patients with known atherosclerosis history and LDL-C ≥ 100 mg/dL (43) receiving atorvastatin (40 mg daily)	MMP-9	Carotid	Serum	Baseline mean carotid TBR values were positively correlated with MMP-9. TBR reduction after the 12-week atorvastatin treatment correlated with changes in MMP-9 levels.	[145]
^18^F-FDG-PET	Participants of dal-PLAQUE trial (130) receiving dalcetrapib	MPO, Lp-PLA_2_	Aorta, Carotid	Plasma	Baseline MPO positively correlated with baseline carotid TBR_mds_. This correlation remained at the 3-month follow-up and was independent of traditional CVD risk factors. Baseline Lp-PLA_2_ mass correlated with aorta TBR_mds_. This correlation disappeared at the 3-month follow-up and was not independent of CVD risk factors.	[149]
^18^F-FDG-PET	Patients underwent CEA for carotid artery stenosis (25)	MMP-9	Carotid	Plaque Tissue	Plaque FDG-PET signal significantly associated with immunohistochemistry MMP-9 content in plaque.	[146]
CT	Patients with PAD stenosis 50–80% (31), PAD stenosis ≥ 80% (22), healthy subjects (27)	VEGF-C, IL-6	Lower limb	Plasma	Serum concentrations of VEGF-C and IL-6 were significantly increased in patients showing moderate or severe peripheral artery stenosis.	[140]
CT	Participants of the SMART study (patients with cardiovascular diseases, 520) and DCS cohort (type 2 diabetes patients, 200)	Inactive Matrix-Gla Protein, osteonectin,	Lower limb	Serum	Inactive matrix-Gla protein was associated with increased MAC prevalence. Osteonectin was associated with decreased risk of MAC compared to IAC.	[168]

In summary, various molecular markers, including those related to inflammation, endothelial dysfunction, oxidative stress, and other processes, have been implicated in PAD pathophysiology. These markers hold promise as diagnostic and prognostic indicators. Several studies have demonstrated correlations between these molecular markers and imaging markers, which further enhanced our understanding of PAD and potentially contributed to developing personalized treatment strategies.

## 9. Challenges and Future Directions

This section discusses the challenges of integrating molecular and imaging markers in assessing PAD and explores potential future directions in the field. These future directions include advancements in imaging technologies and the discovery of novel molecular markers that can enhance our understanding of PAD pathophysiology.

One of the primary challenges in PAD imaging is addressing the limitations of current imaging modalities, such as spatial resolution, sensitivity, and specificity. Accurate characterization of PAD plaques and vessel walls is crucial for a comprehensive understanding of the disease. Additionally, standardizing imaging protocols and quantifying imaging markers for comparison across studies remain significant challenges. For example, MRA has been accused of producing different results across different institutes, limiting its application in clinics and multi-center research [49,59]. Overcoming these technical hurdles will be essential for enhancing imaging modalities’ reliability and clinical applicability in PAD assessment.

In terms of molecular markers, there is a need to identify and validate novel markers that are specific and sensitive to PAD pathophysiology. Unlike carotid and coronary plaques, previous studies found that femoral artery plaque ^18^F-FDG uptake did not correlate with plaque macrophage content and activity, suggesting a unique pathophysiology of PAD and a need for specific markers [116,146]. However, translating molecular markers from preclinical studies to clinical settings presents its challenges. For instance, differences in study populations and methodologies can lead to inconsistencies in the reported associations between molecular markers and PAD. Future research should focus on discovering and validating novel molecular markers and their potential integration with imaging markers to provide a more in-depth understanding of PAD.

Establishing a robust correlation between molecular and imaging markers in PAD is essential for maximizing their diagnostic and prognostic potential. Integrating molecular and imaging data can provide a more comprehensive perspective on the underlying mechanisms of the disease and the factors that contribute to its progression. However, further research is needed to explore the benefits and limitations of this integrative approach, as well as to determine the most effective methods for combining these data sources.

In summary, addressing the challenges associated with integrating molecular and imaging markers in the assessment of PAD is vital for enhancing our understanding of the disease’s pathophysiology. Embracing future directions in this field, such as advancements in imaging technologies and the discovery of novel molecular markers, will be essential for maximizing the potential of these markers in improving PAD patient outcomes. By overcoming these challenges, researchers and clinicians can work towards developing more effective diagnostic and prognostic tools for PAD.

## 10. Conclusions

Significance and Impact: PAD poses a substantial clinical challenge, the prognosis of which depends heavily on effective diagnostic and monitoring methodologies. Current investigations and the available literature demonstrate the importance of non-invasive imaging techniques and molecular markers in evaluating the status and progression of the disease.

Plaque Composition Analysis: Comprehensive understanding of the composition of atheromatous plaques is crucial in PAD management. Techniques such as high-resolution MRI and CT angiography, complemented with the analysis of biomarkers such as inflammatory markers and oxidative stress markers, yield important insights into the nature and characteristics of plaques.

Extent of Atherosclerotic Plaques: Imaging techniques such as CT angiography and MRI provide effective means to quantify the extent of atherosclerotic plaques in PAD patients. Concurrent assessment of molecular markers, especially those indicative of endothelial dysfunction, enriches our understanding by offering a molecular perspective of the ongoing pathological process.

Activity of Atherosclerotic Process: The ability to monitor the activity of the atherosclerotic process is crucial in tracking disease progression and evaluating the effectiveness of therapeutic interventions. The combined application of non-invasive imaging techniques and the analysis of inflammation and oxidative stress markers can provide a reliable indication of the disease activity status.

Significance of Stenosis: The severity of stenosis in PAD is a key determinant of prognosis and therapeutic planning. Precise stenosis evaluation can be achieved with modern imaging modalities such as CT and MRI. Combined with biomarker analysis, these tools can provide clinicians with a robust basis for predicting outcomes and formulating individualized treatment strategies.

Future Perspectives: The future holds promising advancements in non-invasive imaging and molecular marker research, with potential benefits for PAD management. Emerging techniques such as PET and SPECT, along with novel molecular markers, are set to enhance our understanding of the disease and its management.

Integrating non-invasive imaging techniques with molecular markers is vital for comprehensively understanding and managing PAD. It is our hope that the ongoing advancements in this field will continue to improve our understanding of the pathophysiological processes underlying PAD, ultimately resulting in improved patient care.

## Figures and Tables

**Table 1:** Advantages and disadvantages of different modalities for imaging PAD plaques.

Imaging Modalities	Advantages	Disadvantages
CT	High spatial resolution. Fast acquisition time. Widespread availability. Ability to visualize arterial stenosis, calcification, and plaque morphology.	Exposure to ionizing radiation. Potential need for iodinated contrast agents. Limited soft-tissue contrast.
MRI	Excellent soft-tissue contrast. No ionizing radiation. Can be conducted with or without contrast. Ability to visualize vessel walls and plaque components.	Inability to accurately visualize calcification. Longer scanning times. Potential susceptibility to artifacts. Contraindications for certain patients (e.g., some MR incompatible implants).
Nuclear Imaging (PET and SPECT)	Visualization of molecular and cellular processes. In vivo assessment of plaque metabolism, inflammation, and neovascularization.	Use of ionizing radiation. Lower spatial resolution compared to CT and MRI. Need for specialized equipment and radiotracers. Long scan time depending on radiotracer.

## Data Availability

Not applicable.
